# Sensory Loss and Cognitive Decline among Older Adults: An Analysis of Mediation and Moderation Effects of Loneliness

**DOI:** 10.1093/geroni/igaa057.3399

**Published:** 2020-12-16

**Authors:** Shaoqing Ge, Wei Pan, Bei Wu, Brenda Plassman, XinQi Dong, Eleanor McConnell

**Affiliations:** 1 University of Washington School of Nursing, Seattle, Washington, United States; 2 Duke University, Durham, North Carolina, United States; 3 New York University, New York, New York, United States; 4 Rutgers University, New Brunswick, New Jersey, United States

## Abstract

Multiple studies have reported that hearing and vision loss are linked to cognitive decline. Yet little is known about factors that may influence the association between sensory loss and cognitive decline. This study examined if loneliness mediates or moderates the impact of sensory loss on cognitive decline as individuals age. This was a longitudinal study using data (N = 243) from the Health and Retirement Study (HRS) (2006 – 2014) and its supplement: The Aging, Demographics, and Memory Study (ADAMS) (Wave C). Hearing loss was defined by an inability to hear pure-tone stimuli of 25 dB at frequencies between 0.5 – 4.0 kHz in either ear. Vision loss was defined as having corrected binocular vision worse than 20/40. Loneliness was measured by the 3-item UCLA Loneliness Scale. Longitudinal parallel-process (LPP) analysis was conducted at a significance level of α = .05 (one-tailed). Loneliness moderated but did not mediate the associations between vision loss and the rate of cognitive decline (standardized β = -.108, p < .05). No moderation or mediation effect of loneliness was found for the association between hearing loss and cognitive decline. Both hearing and vision loss were significantly associated with increased severity of loneliness. Vision loss combined with an elevated level of loneliness may produce a more synergistic, deleterious impact on older adults’ cognitive function than vision loss alone. This study highlights the importance of promoting a healthy social and psychological status for older adults with vision loss.

